# Combined lung-kidney transplantation: First case in Portugal

**DOI:** 10.1016/j.rmcr.2021.101386

**Published:** 2021-03-19

**Authors:** David T. Silva, Carolina Dantas, Ana Sofia Santos, Cecilia Silva, Inês Aires, Francisco Remédio, Sofia Carrelhas, Ana Pena, João Eurico Reis, Paulo Calvinho, Luísa Semedo, João Cardoso, Fernando Nolasco, José Fragata

**Affiliations:** a Pulmonology Department, Hospital of Santa Marta, Centro Hospitalar Universitário Lisboa Central (CHULC), Portugal; b Cardiothoracic Department, Hospital de Santa Marta, Centro Hospitalar Universitário Lisboa Central (CHULC), Portugal; c Nephrology Department, Hospital Curry Cabral, Centro Hospitalar Universitário Lisboa Central (CHULC), Portugal; d General Surgery Department, Hospital Curry Cabral, Centro Hospitalar Universitário Lisboa Central (CHULC), Portugal; e NOVA Medical School, Faculdade de Ciências Médicas, Universidade Nova de Lisboa, Portugal

**Keywords:** Combined lung-kidney transplantation, Vv-ecmo, Cystic fibrosis, Continuous venovenous hemodiafiltration

## Abstract

A significant dysfunction of another organ is usually considered an absolute contraindication for lung transplantation, unless multiorgan transplantation is indicated and practical, as is the case of combined lung-kidney transplantation. Few cases of combined lung-kidney transplantation have been described in the literature; however, it is known that, in certain cases, it is the only way to offer an opportunity to selected patients with renal and lung dysfunction. The authors are not aware of any previously published case of a patient receiving both extracorporeal membrane oxygenation and continuous venovenous hemodiafiltration as a bridge for combined kidney-lung transplantation.

The authors present the first case of combined lung-kidney transplantation performed in Portugal.

## 1 Case report

**Fig. A1:**
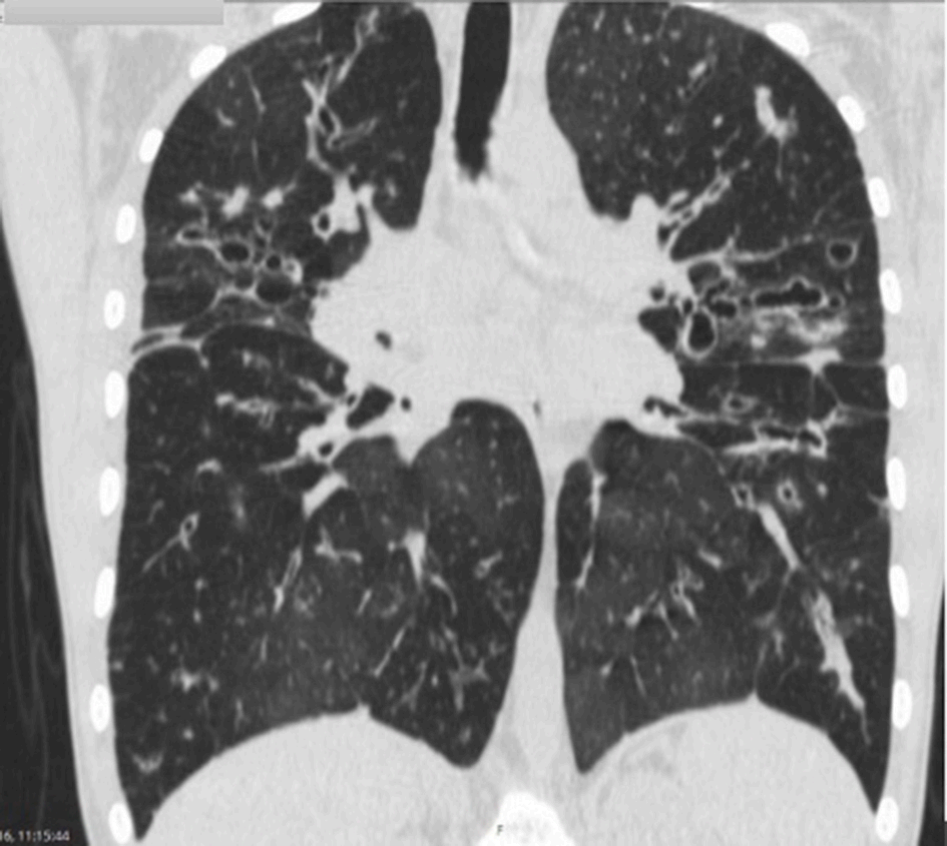
Coronal plane of computerized tomography in June 2016.

**Fig. A2:**
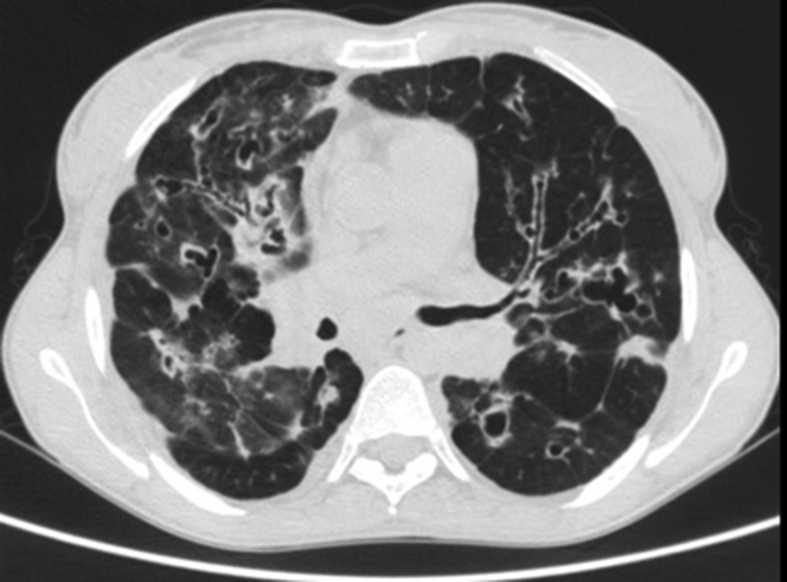
Axial planes of computerized tomography in June 2016.

The authors present the case of a 29-year-old woman, diagnosed with cystic fibrosis at the age of 3 (homozygous for F508del), with exocrine pancreatic insufficiency and pansinusitis. She has been monitored as a pre-transplantation consultation since June 2016, with extensive cystic bronchiectasis (Fig. A1 and A2) with *Pseudomonas aeruginosa* and *Aspergillus fumigatus* colonization, as well as multiple hospitalizations for respiratory infections. At the time of admission, she weighed 52 kg was 165 cm tall and had Forced Expiratory Volume in 1 second (FEV1) of 720 cc (21%) and Total Lung Capacity (TLC) of 3650 cc (79%). She was also hypoxemic, requiring oxygen support therapy. The patient entered the list for lung transplantation in February 2017, with low priority, due to her clinical and functional stability. For better understanding, we perform a timeline (Table 1).

**Table 1:**
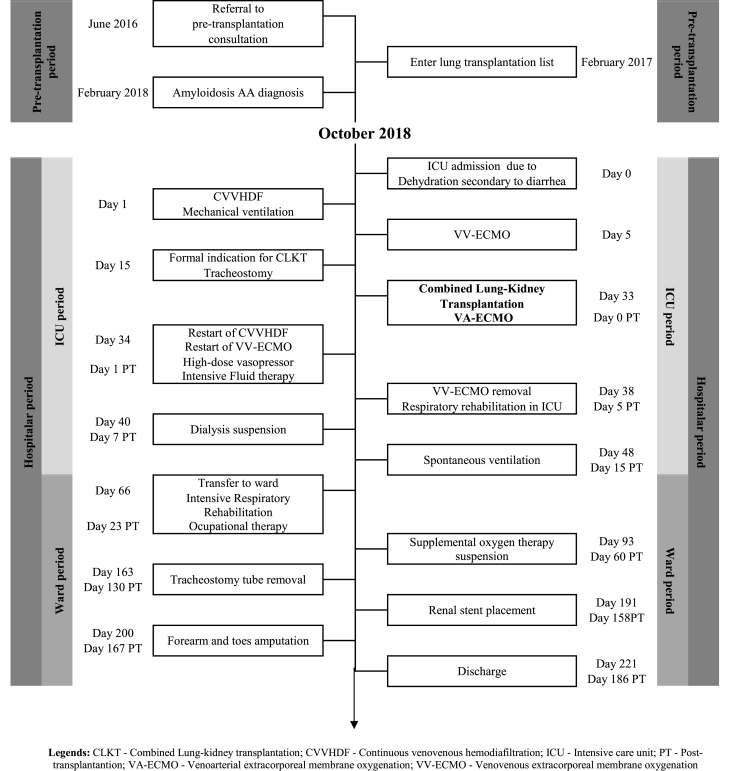
Timeline.

**Legends:** CLKT - Combined Lung-kidney transplantation; CVVHDF - Continuous venovenous hemodiafiltration; ICU - Intensive care unit; PT - Post-transplantantion; VA-ECMO - Venoarterial extracorporeal membrane oxygenation; VV-ECMO - Venovenous extracorporeal membrane oxygenation.

**Fig. B1:**
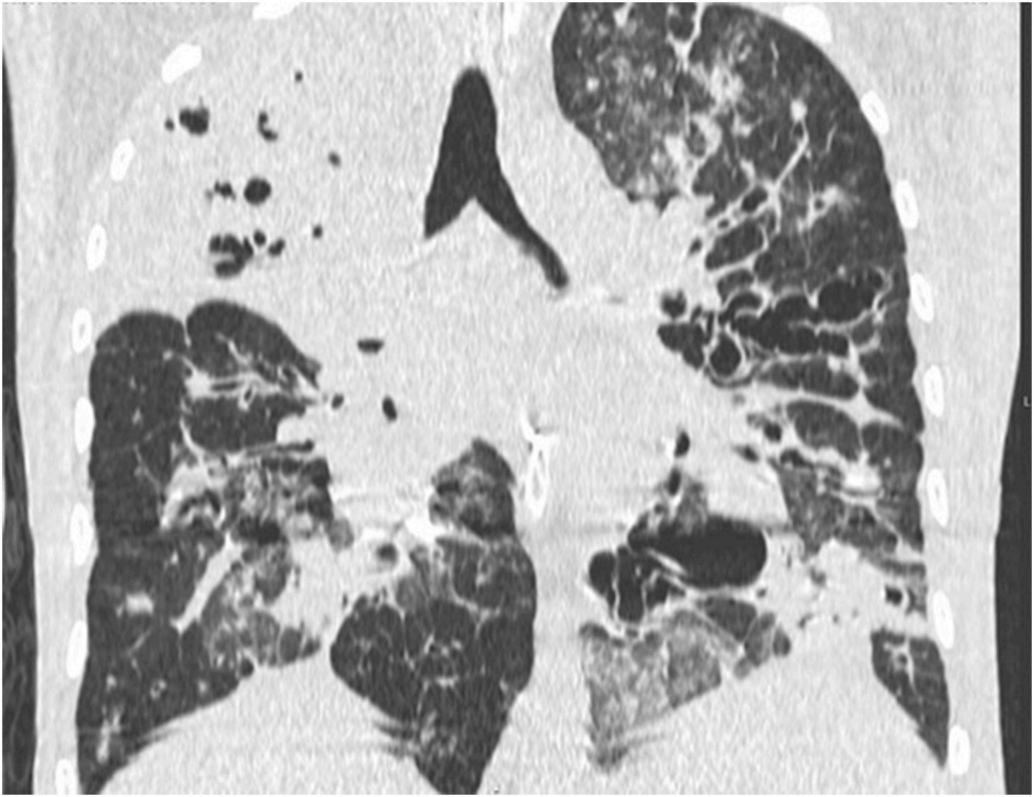
Coronal plane of computerized tomography at the time of ICU admission in October 2018.

**Fig. B2:**
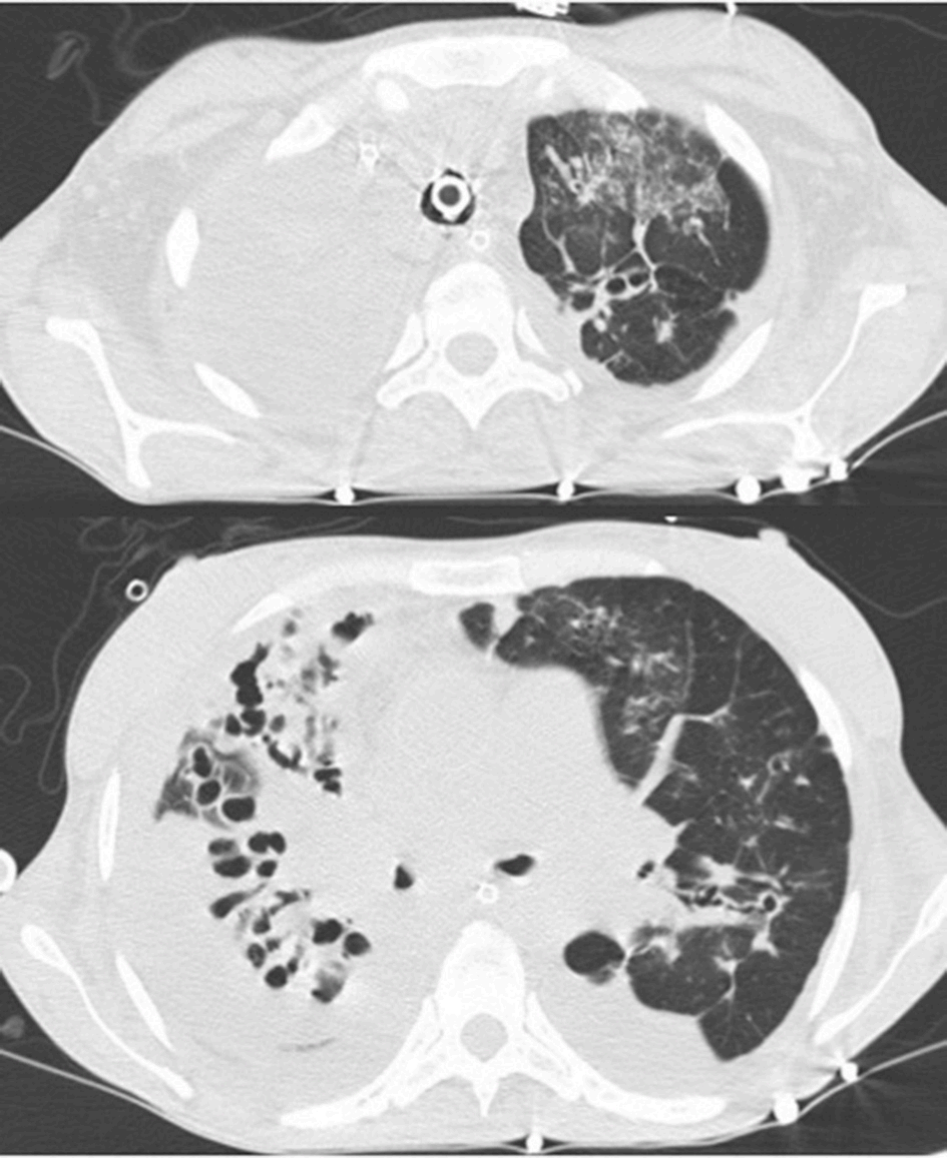
Axial planes of computerized tomography at the time of ICU admission in October 2018.

To investigate the appearance of nephrotic proteinuria in February 2018, she underwent a renal biopsy that revealed deposits of amyloid A, and the diagnosis of AA amyloidosis (or secondary amyloidosis) was established. In October 2018, the patient was admitted to the Intensive Care Unit (ICU) for dehydration secondary to diarrhea, with subsequent acute renal failure and oligoanuria requiring continuous venovenous hemodiafiltration (CVVHDF). At the ICU, she developed a respiratory infection (Fig. B1 and B2) and septic shock requiring invasive mechanical ventilation. Antibiotic therapy was started with ceftolozane/tazobactam and ciprofloxacin considering an antibiotic sensitivity test (AST) positive for *Pseudomonas aeruginosa* infection. Due to worsening respiratory failure, it was necessary to start venovenous extracorporeal membrane oxygenation (VV-ECMO). After failure of weaning from mechanical ventilation, the patient underwent tracheostomy, maintaining invasive mechanical ventilation, awake-ECMO and CVVHDF. The absence of improvement in renal function granted formal indication for combined lung-kidney transplantation (CLKT).

During the 32 days of ICU stay, 27 of them under “awake-ECMO,” the patient suffered hemothorax and hemopericardium secondary to anticoagulation, which evolved to cardiac arrest that was successfully reversed. Physical rehabilitation was limited at this point, and only in-bed mobilization exercises were feasible.

On the 33rd day of the ICU stay, the patient was underwent CLKT. The donor was a 69-year-old woman, weighing 60 kg and measuring 171 cm, with no relevant medical history. She had a predicted TLC of 4050 cc. Her last arterial blood gas analysis had pO_2_ of 512 mmHg with FiO_2_ of 1.0 and a positive end-expiratory pressure of 5 cmH_2_O for 5 min. Tracheobronchial aspirate cultures were negative. The donor explants were performed in a multiorgan context without complications, and the lungs were preserved in Perfadex®.

A sequential double-lung transplant was performed under venoarterial ECMO. It was very laborious because of the existence of bilateral empyema, which led to extrapleural pneumectomy with profuse hemorrhage, due to the patient's hypocoagulant status. In total, one prothrombin complex, 15 red cell concentrates, five platelet concentrates and four fresh frozen plasma units were required to support the patient. Ischemic times were 6 h 18 min for right lung and 8 h 13 min for left lung. After lung transplantation, kidney transplantation was performed, with an ischemic time of 11 h 57 min and no major complications noted, and good perfusion was observed after implantation.

The immediate post-transplant period was complicated by multifactorial shock, requiring the restart of CVVHDF, high-dose vasopressor support (noradrenaline up to 2 μg/kg/min and dopamine up to 4 μg/min), VV-ECMO and intensive fluid therapy.

Immunosuppression included induction with 20 mg basiliximab on day 0 and day 4 of transplantation and maintenance therapy with tacrolimus, which was started during surgery (target blood levels around 10 ng/mL in the first 6 months and 6 ng/mL afterwards), mofetil mycophenolate and steroids. Corticosteroid therapy was started during the transplant, at the unclamping of each pulmonary artery, initially with methylprednisolone 500 mg I.V., continuing with the following weaning schedule: 125 mg tid on day 1, 125 mg bid on day 2, and 125 mg qd on day 3, with weaning of 10 mg per day down to a daily dose of 0.5 mg/kg/day prednisolone for the first month after transplant and progressive weaning down to 0.1 mg/kg/day after the first month.

She was started on empirical antibiotics with meropenem, as well as anidulafungin directed at *Candida albicans* and *C. dubliniensis* (isolated in the recipient's bronchoalveolar lavage) and to previous isolations of *A. fumigatus*. Prophylaxis with inhaled colistin and prophylactic therapy for cytomegalovirus with ganciclovir (switched to valganciclovir at the end of the 14th day) were also carried out.

There was slow improvement and complicated ventilatory weaning due to marked sarcopenia, which also motivated the assessment of renal function based on serum cystatin C rather than creatinine levels, estimating a glomerular filtration rate of 25–35 mL/min. The patient was subsequently submitted to amputation of the left forearm, fourth and fifth toes of the left foot and distal phalanx of the fifth toe of the right foot due to irreversible ischemia, owing to the high aminergic support in the pre-operative, intra-operative and post-operative periods. In the first five days post-transplantation, rehabilitation exercises aimed to improve orthostatic positioning and passive limb mobilization.

The discontinuation of VV-ECMO was only possible on day 5 post-transplant, while continuous dialysis was suspended on day 7 post-transplant. After this, she showed progress in the physical rehabilitation program, and started resistance training with light weights, postural re-education and transfer to chair. Spontaneous ventilation was achieved on day 50, supplemental oxygen therapy was suspended on day 60 and tracheostomy was removed on day 130 post-transplant.

During her stay in the Transplant Unit, the patient also underwent multiple cycles of antibiotics for nosocomial infections, in addition to those previously described, namely 7 days of tigecycline directed at *Klebsiella oxytoca* isolated from a renal graft; 21 days of ceftazidime-avibactam and intravenous colistine, respectively, directed at *Pandoraea* and *P. aeruginosa* isolated in pleural fluid; fluconazole directed at *C. parapsilosis* in urine; and 15 days of cotrimoxazole and ceftriaxone to treat *K. pneumoniae* sepsis of urinary origin.

Transfer to the Pulmonology ward took place on the 23rd day post-transplant. Serial renal Doppler ultrasonography was performed, which showed renal artery stenosis requiring stent placement (day 158 post-transplant).

At the ward, it was possible to start an intensive rehabilitation program and occupational therapywith progressive gain of autonomy. The patient continued to perform resistance training with light weights, stretching and started to walk in the corridor. Only after the first 20 days in the ward, she did undergo respiratory and physical rehabilitation in the gym, where she started to do resistance training with progressively heavier weights, chest wall mobility and chest expansion exercises and walking on treadmill, with progressively higher speed settings. No additional complications, renal, pulmonary or otherwise, were identified.

The patient was discharged from the hospital 186 days after the transplant and after 224 days of hospitalization, with FEV1 of 1790 cc (54%), FVC 2030 cc (52%) and DLCO 56% (KCO 84%), creatinine 2.31 mg/dL, blood urea nitrogen 100 mg/dL, and cystatin C 2.64 mg/L, with an estimated GFR of 22 mL/min.

She maintains follow-up on the kidney and lung transplant services. Twenty-four months after CLKT, the patient wears a forearm prosthesis. She denies any major complaints and presents with FEV1 2520 cc (74%), FVC 3420 cc (85%), creatinine 1.4 mg/dL, and blood urea nitrogen 38 mg/dL, with an estimated GFR of 50 mL/min.

## 2 Discussion

The hospital of Santa Marta, in Lisbon, has been the lung transplantation center of reference in Portugal since 1991, when the first combined heart-lung transplant was performed. It was only in 2001 that the first isolated lung transplantation took place. Twenty-one lung transplants were performed before 2008, when the team was reorganized and new protocols were elaborated. Since then, more than 300 lung transplantation have been performed, but, to date, no CLKT has never been carried out.

In Portugal, cystic fibrosis is only the third leading cause of lung transplantation [1], and worldwide, it is the third most common cause for CLKT [2]. Although lung involvement is more characteristic, cystic fibrosis can also affect the pancreas, liver, kidneys and intestine. The evolution of the disease can be very variable; however, recurrent respiratory infections are common, which become more frequent throughout life. In the presented case, the successive infectious events promoted the deposition and accumulation of amyloid A protein at the renal level, with dysfunction and consequent renal failure.

The combined transplantation of solid organs has evolved in recent years, however, with few studies carried out [3].

Regarding the CLKT, particular attention should be paid to the type and dose of immunosuppression used in order to reduce the nephrotoxic effects and the risk of infection as to not compromise the viability of the pulmonary and renal grafts, especially taking into account the use of calcineurin-inhibiting drugs. In this case, it was tacrolimus, and the main indication for kidney transplantation in lung transplant patients is the nephrotoxicity caused by this type of immunosuppressant [4]. On the other hand, the detailed control of water balance is fundamental in the management between risk of renal dysfunction due to dehydration and the risk of pulmonary edema due to excess fluid accumulation. According to Reich et al. [3] and Cassuto et al. [5], patient survival after CLKT was similar to isolated lung transplantation, and Otani et al. [6] showed that CLKT significantly increased life expectancy, preserved lung function, and improved the quality of life, which suggests that CLKT is a feasible therapeutic option for lung transplantation candidates with significant renal dysfunction.

The authors would like to highlight the extreme complexity of the patient and the extreme severity of her clinical condition at the time of the transplant and in the following weeks that she was hospitalized. In fact, it was the first time in our center that a patient has undergone a transplant and has been on VV-ECMO, CCVDHF and invasive mechanical ventilation concurrently and for so long. The recipient's youth, as well as the good conditions of the pulmonary graft, together with an experienced multidisciplinary team, certainly helped in the successful outcome of this case.

In conclusion, CLKT may be indicated in carefully selected lung transplantation candidates with end-stage renal disease, as long as it is possible to proceed with kidney transplantation, ideally at the same operative time.

## Declaration of competing interest

All authors report no conflict of interest.
